# Supplementary figures and images for: One-shot phase-recovery using a cellphone RGB camera on a Jamin-Lebedeff microscope
Source: PLoS One. 2019 Dec 31;14(12):e0227096. doi: 10.1371/journal.pone.0227096 (PMC6938357; doi:10.1371/journal.pone.0227096)

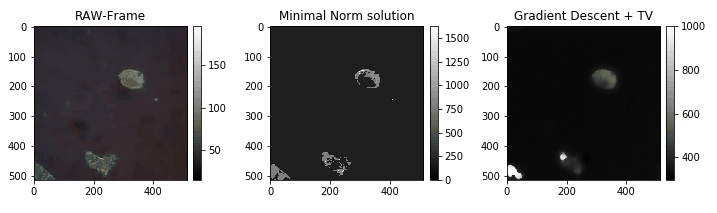

Supplement: S1 Video — In this video we give a comparison of raw RGB data, the minimal-norm solution and the TV-regularized iterative algorithm of a live-cell image series with a framerate of 30 frames per second (fps). (GIF) [file pone.0227096.s001.gif]

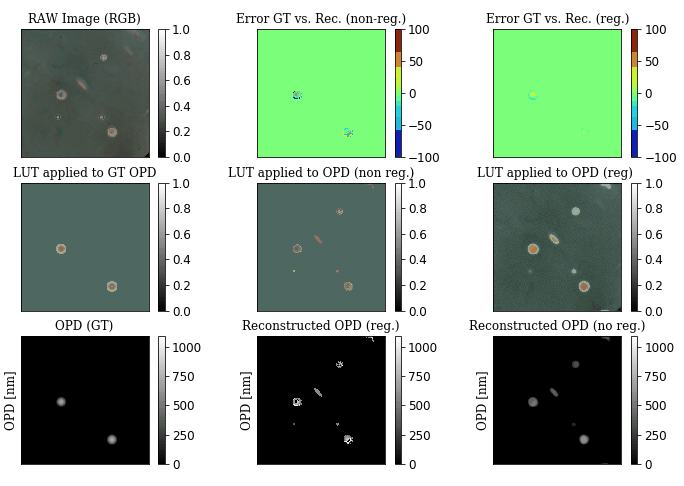

Supplement: S2 Video — The graphs give the percentaged error between the expected ground-truth measurements and the reconstruction using regularized and non-regularized computations of the OPD. (GIF) [file pone.0227096.s002.gif]
