# Supplementary material for: One-shot phase-recovery using a cellphone RGB camera on a Jamin-Lebedeff microscope
Source: PLoS One. 2019 Dec 31;14(12):e0227096. doi: 10.1371/journal.pone.0227096 (PMC6938357; doi:10.1371/journal.pone.0227096)
Supplement: Attachment — Submitted filename: Plos response to reviewers.pdf [file pone.0227096.s003.pdf]

Leibniz Institute of Photonic Technology
Albert-Einstein Straße 9, 07745 Jena, Germany
+49 3641 206 434
rainer.heintzmann @leibniz-ipht.de

Rainer Heintzmann

Dear editor,

We would like to thank the reviewers for their comments and suggestions for the manuscript. We took the concerns and suggestions raised in the review into account for further enhancements in the general method and experiment design. The suggestions identified important areas which required improvements and increases the quality of the presented results.

Additionally we followed the concerns about the video-file and add it as a ".gif" which is readable by most computers.

Below, you will find a point by point description of how each comment was addressed in the manuscript. Original reviewer comments in boldface, responses in regular typeface.

Comments to the Author

1. Is the manuscript technically sound, and do the data support the conclusions?

The manuscript must describe a technically sound piece of scientific research with data that supports the conclusions. Experiments must have been conducted rigorously, with appropriate controls, replication, and sample sizes. The conclusions must be drawn appropriately based on the data presented.

Reviewer #1: Partly
Reviewer #2: Yes

Further experiments were done to show the long-term accuracy of the phase recovery using the proposed method.

---

2. Has the statistical analysis been performed appropriately and rigorously?

Reviewer #1: N/A
Reviewer #2: I Don't Know

We added a statistical analysis to show the long-term accuracy of the phase recovery using the proposed method on a cellphone. All results have also been shared on the github website.

---

3. Have the authors made all data underlying the findings in their manuscript fully available?

The PLOS Data policy requires authors to make all data underlying the findings described in their manuscript fully available without restriction, with rare exception (please refer to the Data Availability Statement in the manuscript PDF file). The data should be provided as part of the manuscript or its supporting information, or deposited to a public repository. For example, in addition to summary statistics, the data points behind means, medians and variance measures should be available. If there are restrictions on publicly sharing data—e.g. participant privacy or use of data from a third party—those must be specified.

Reviewer #1: Yes

Reviewer #2: Yes

---

4. Is the manuscript presented in an intelligible fashion and written in standard English?

PLOS ONE does not copyedit accepted manuscripts, so the language in submitted articles must be clear, correct, and unambiguous. Any typographical or grammatical errors should be corrected at revision, so please note any specific errors here.

Reviewer #1: Yes
Reviewer #2: Yes

---

5. Review Comments to the Author

Please use the space provided to explain your answers to the questions above. You may also include additional comments for the author, including concerns about dual publication, research ethics, or publication ethics. (Please upload your review as an attachment if it exceeds 20,000 characters)

Reviewer #1: The authors suggest using Jamin-Lebedeff (JL) polarization interference microscopy for quantitative phase microscopy of biological cells. The optical path difference (OPD) between the sample and the reference, which should be empty, is encoded into a colour by white-light interference. Then, a colour-table is used to calculate the OPD map. The paper uses a phase retrieval method relying on a single-shot measurement, allowing real-time quantitative phase imaging. The system includes a cellphone with an RGB camera.

The problem in the paper is that there are not enough experimental results, which are quantified in respect to a ground truth. Cells might come in different shapes and heights. I think you should add imaging of known diameter microscopic beads (5-10 micron), and also add a quantitative analysis that gives the values of the temporal and spatial standard deviations of the obtained OPD map.

The reviewer is rising a very important point about the accuracy of the recovered phase measurements. Therefore, we introduced an additional paragraph addressing this issue. To have proper ground-truth data, we took a set of polystyrene microspheres and immersed them in oil with known refractive index. This produces samples with a distinct optical path difference which can be modelled artificially in the computer and compared with the experimental data. We show, that the regularized phase-recovery is superior over the minimal norm solution across many time-points (i.e. video-frames).

Finally, the video does not run. Please submit in a different format.
We have tested the video-file using VLC (videolan.org) which seemed to work. Nevertheless, we thank the reviewer to point out the incompatible file-format. We added the files in the more general ".gif" format.

Reviewer #2: To meet the need of quantitative phase measurements, a cellphone RGB camera is used to capture image from a Jamin-Lebedeff microscope and look-up-table (LUT) is created to convert the measured RGB signal of a phase-sample into an OPD. The method is straightforward, but the accuracy of phase measurement is uncertain.

This point reflects the opinion of Reviewer #1 and is an important aspect in order to improve the quality of this manuscript. We have added a generous accuracy analysis over multiple video-frames and show that our algorithm has a general mean-error of 12 +/- 5% compared to the pure minimal-norm solution of 24 +/-2 % which is majorly caused by the phase-wraps in the recovered images.

While the smart phone can compute the phase directly, it needs extra effort to get a good images than using the regular microscope camera directly. The contribution to demonstrated application is very limited.

We want to emphasize, that this method can also be used with any RGB camera with the same effort. Since the recovered phase only depends on a reference measurement, the recovery-procedure is the same for any RGB camera (including generic industry-grade CMOS sensors.). Thanks for the reviewer, that this point was not clearly written. We have add this to the manuscript.

Editor:
The revisions relates to:
1. Further phase measurements which are quantified and compared with respect to 'ground truth' phase object,
2. Quantitative analysis of the temporal and spatial standard deviations of the obtained OPD map.

Thank you very much for summarizing the issues aroused by the reviewers. We did a generous analysis of long-time image acquisition with ground-truth data which can be found in the manuscript and in the newly attached Supplementary video.

Please do not hesitate to contact me as a correspondence author of the presented manuscript.

With many thanks in advance for your attention,

Very sincerely yours,
Rainer Heintzmann
